# Case Report: *Pseudomonas* liver abscess in a previously healthy child with homozygous pathogenic S allele variant of the *SERPINA1* gene

**DOI:** 10.3389/fimmu.2026.1755963

**Published:** 2026-03-27

**Authors:** Sanya Thomas, Alaric W. D’Souza, Musaab Alhezam, Craig D. Platt, Ofer Levy

**Affiliations:** 1 Precision Vaccines Program, Department of Pediatrics, Boston Children’s Hospital, Boston, MA, United States; 2 Department of Pediatrics, Harvard Medical School, Boston, MA, United States; 3 Division of Infectious Diseases, Department of Pediatrics, Boston Children’s Hospital, Boston, MA, United States; 4 Department of Immunology and Infectious Diseases, Harvard T.H. Chan School of Public Health, Boston, MA, United States; 5 Division of Immunology, Department of Pediatrics, Boston Children’s Hospital, Boston, MA, United States; 6 Global Health Initiative, Broad Institute of MIT & Harvard, Cambridge, MA, United States

**Keywords:** alpha-1 antitrypsin, case report, liver abscess, *Pseudomonas*, serine protease inhibitor, SERPINA1

## Abstract

**Background:**

Pyogenic liver abscess is an uncommon pediatric condition, and *Pseudomonas aeruginosa* represents a rare causative agent in otherwise healthy children. Such infections may signal an underlying inborn error of immunity. Alpha-1 antitrypsin (A1AT), encoded by the *SERPINA1* gene, is a serine protease inhibitor with key immunomodulatory functions. In mouse models, A1AT prevents degradation of the antimicrobial SPLUNC1 protein to enhance host defense against *Pseudomonas aeruginosa*. In humans, while A1AT variants can be associated with a generally increased risk of respiratory infection, to our knowledge no corresponding impact with respect to *P. aeruginosa* has been reported.

**Case Description:**

We report a previously healthy 14-month-old girl who developed *P. aeruginosa* liver abscess without identifiable predisposing factors. Comprehensive immunologic evaluation was unremarkable except for whole exome sequencing revealing homozygous S allele pathogenic *SERPINA1* variant (c.863 A>T, p.E288V) known as the S allele associated with partial A1AT deficiency. The clinical presentation, microbiologic findings, and disease course suggested that defective A1AT function may have contributed to dysregulated inflammation and susceptibility to infection.

**Conclusion:**

This case highlights a potential association between homozygosity in the pathogenic S variant of *SERPINA1* and susceptibility to severe *P. aeruginosa infection*, in this case liver abscess. While A1AT deficiency is classically associated with pulmonary and hepatic disease, the immunomodulatory role of A1AT suggests broader relevance in host defense. Early recognition of underlying *SERPINA1* defects in children with unusual or severe infections may inform prognosis, guide management, and prompt appropriate genetic counseling and surveillance for long-term complications.

## Introduction

Liver abscess is an uncommon infection in children (1). *Pseudomonas aeruginosa* is a rare cause of pyogenic liver abscess in otherwise healthy pediatric patients, accounting for ~2-6% of cases (2, 3). Inborn errors of immunity (IEI) may predispose affected children to such infections.

Alpha-1 antitrypsin (A1AT) is a plasma serine protease inhibitor (SERPIN) primarily synthesized by hepatocytes. As an acute phase glycoprotein, A1AT primarily inhibits proteinase 3 and neutrophil elastase (NE), enzymes involved in bacterial clearance and activation of pattern recognition receptors such as Toll-like receptors. Through these mechanisms, A1AT modulates the production of pro-inflammatory cytokines and exerts both immunoregulatory and anti-inflammatory effects on innate and adaptive immune cells (4–9). Although A1AT can transiently promote pro-inflammatory cytokine production, its long-term effect is predominantly anti-inflammatory, and it modulates neutrophil function – neutrophils being critical for host defense against *Pseudomonas* (10–12).

A1AT is encoded by the *SERPINA1* gene located on the long arm of chromosome 14, and it exhibits high polymorphism. The M allele represents the functional wild-type variant, whereas the Z and S alleles are associated with A1AT deficiency (A1ATD), leading to polymerization and intracellular accumulation of A1AT in hepatocytes, subsequently resulting in liver injury (13–15). As an acute phase reactant, A1AT expression is enhanced by inflammatory cytokines such as IL-6 (16). Normal plasma concentrations average 1.3 g/L (reference range: 0.9–2 g/L) with a half-life of 4–5 days (4).

Here, we describe a case of *Pseudomonas aeruginosa* liver abscess in a previously healthy child found to be homozygous for the S allele pathogenic p.Glu288Val variant in *SERPINA1*. We hypothesize that the defective *SERPINA1*-encoded A1AT may have impaired host defense mechanisms against *P. aeruginosa* infection.

## Case description

### Clinical description

A 14-month-old female presented with high-grade fever (Tmax 40 °C), irritability, poor oral intake, and reduced urine output for one week. She was treated with oral amoxicillin for otitis media in the setting of persistent febrile illness and symptoms associated with upper respiratory tract infection. Three days later, she presented to the emergency department with vomiting, diarrhea, and reduced oral intake. She tested positive for respiratory syncytial virus (RSV) and rhinovirus, and over the next 24 hours, appeared dehydrated with anuria, generalized edema, and abdominal distension. She was admitted for further evaluation. Her hospital course is summarized in **Table 1**.

**Table 1:** Hospital course.

Hospital day	Event
-5 → -1	**Initial admission to outside hospital** Presenting symptom: Fever Labs: WBC = 5.1 K cells/µL, ANC = 900 cells/µL, Hb = 8.4 g/dL, albumin = 3.1 g/dL, CRP = 31.1 mg/dL, ESR = 56 mm/hr Ultrasound abdomen: mild ascites, mild splenomegaly Treatment: Ceftriaxone (empirical)
0	**Transferred to BCH** Findings: persistent fever, worsening edema, hypoalbuminemia, anemia, abdominal distension Labs: albumin = 2.5 mg/dL, Hb = 7.2 g/dL, elevated inflammatory markers (CRP = 38.7 mg/dL, procalcitonin = 39.1 ng/mL, elevated ESR) Microbiology: Nasopharyngeal swab PCR RSV+, rhinovirus+ Empiric antibiotics: Cefepime, metronidazole, vancomycin Symptomatic management: diuretics, albumin replacement, PRBC transfusions
10	Transitioned to cefepime monotherapy after initial broad-spectrum coverage
27	Initiation of GCSF (Filgrastim) 50 µg daily
35	Discharged on ciprofloxacin monotherapy

WBC, white blood count; ANC, absolute neutrophil count; Hb, hemoglobin; CRP, C-reactive protein; ESR, erythrocyte sedimentation rate; BCH, Boston Children’s Hospital; RSV, respiratory syncytial virus; PCR, polymerase chain reaction; PRBC, packed red blood cells; GCSF, granulocyte colony stimulating factor.

### Medical and family history

She was born at full term and was appropriately vaccinated for age. She had presumed milk protein allergy in infancy, managed with an amino acid-based formula. Cow’s milk was reintroduced two weeks prior to presentation. She had no history of severe, atypical, or recurrent infections or hospitalizations. Her mother reported contact with a cousin with vomiting and diarrhea ~3 days before her initial symptoms. There was no known family history of inborn errors of immunity. Her mother had a history of thyroid disease, and her paternal grandmother was diagnosed with Crohn’s Disease at 30 years of age.

### Physical examination

On examination, she was alert, febrile, and appeared irritable and tired. She had rhinorrhea, nasal congestion, dry mucous membranes, and was tachycardic (pulse rate: 168/minute). Her capillary refill time was 3 seconds. Other vital signs were within normal limits (blood pressure: 106/76 mmHg, respiratory rate: 60/minute). She had bilateral 2+ pitting edema in her hands and lower extremities. On systemic examination, her abdomen was distended and tender to palpation, but without rebound tenderness or guarding. There was no fluid wave, shifting dullness, or mass palpable per abdomen. The remainder of her physical exam was unremarkable.

### Diagnostic assessment

**Figure 1:**
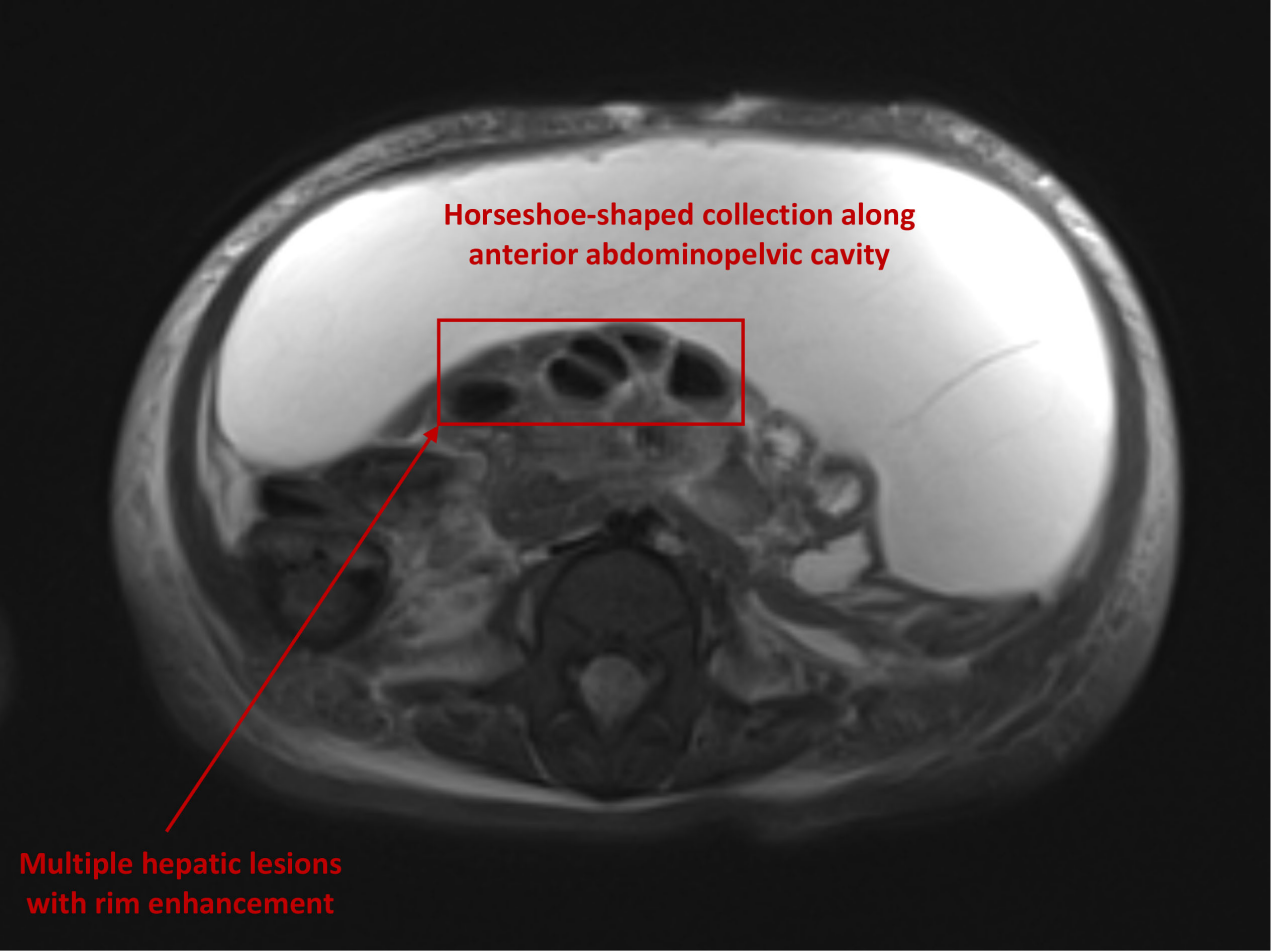
Liver abscesses and ascites on abdominal MRI.

Abdominal MRI (**Figure 1**) revealed multiple hepatic lesions with rim enhancement suggestive of liver abscesses. There was mild to moderate edema within the liver parenchyma surrounding the lesions. A large horseshoe-shaped collection along the anterior aspect of the abdominopelvic cavity was observed in keeping with the loculated ascites. Small amounts of bilateral perirenal fluid, bibasilar atelectasis and pleural effusions (left>right), and soft tissue anasarca were noted.

Trio rapid genome sequencing (GenomeXpress) was obtained in the setting of her liver abscesses, systemic inflammation, and protein-losing enteropathy. There were no candidate variants among genes associated with known IEI. However, a homozygous pathogenic S allele variant was found in *SERPINA1* (c.863 A>T, p.E288V) known to cause partial A1ATD. Her father was also homozygous for the p.E288V variant, and her mother was heterozygous for this variant. Serum A1AT levels were normal. Liver enzymes and cholestatic parameters were normal. These laboratory and radiographic data are summarized in **Table 2**. A comprehensive immunologic evaluation was undertaken (**Table 3**) including immunoglobulin levels, vaccine antibody responses, lymphocyte subsets, complement activity, and neutrophil oxidative burst testing, all of which were normal. Her peripheral blood smear did not demonstrate any features suggestive of neutrophil-specific granule deficiency.

**Table 2:** Laboratory and radiographic data.

Laboratory / radiological investigations	Outside hospital	Boston children’s hospital	Normal ranges
Hemoglobin (g/dL)	**8.4**	**7.2** ^1^	11.0–13.5
WBC (1000 cells/µL)	5.1		5.90–14.88
ANC (cells/µL)	**900**	**0.09** **0.56**	1.47–4.83
Serum albumin (g/dL)	3.1	**2.5**	3.0–4.6
INR		**1.4**	0.93–1.15
ESR (mm/hr)	56	**elevated**	<30
CRP (mg/dL)	**31.1**	**38.7** **4.99**	<0.50
Procalcitonin (ng/mL)		**39.1**	<0.1
A1AT (mg/dL)		180	76-190
ALT (unit/L)		18	3-30
GGT (unit/L)		8	8-35
Alkaline phosphatase (unit/L)		142	110-400
LDH (U/L)		193	110-295
Total bilirubin (mg/dL)		**0.2**	0.3-1.2
Direct bilirubin (mg/dL)		0.1	<= 0.4
Viral antigen tests		**RSV and rhinovirus positive** (nasopharyngeal swab) Viral PCR, GI PCR^2^, Toxoplasma, Bartonella: Negative	
Blood culture		No growth	
Urine culture		No growth	
Paracentesis		***Pseudomonas aeruginosa*** (pan-susceptible)	
Liver biopsy		Moderate panlobular macrovesicular steatosis; focal mild portal chronic inflammation; CK7 immunostain highlighted focal ductular reaction; iron stain highlighted iron deposition in predominantly Kupffer cells; PAS-D stain highlighted ceroid-laden macrophages; no PAS-positive, diastase-resistant cytoplasmic globules seen; no significant fibrosis seen	
Liver abscess aspirate		***Pseudomonas aeruginosa*** (pan-susceptible)	
Liver abscess biopsy		Portion of abscess with acute inflammation and necroinflammatory debris with Gram-negative rods seen on Gram stain; adjacent mixed acute, chronic, and histiocytic inflammation; moderate panlobular macrovesicular steatosis; no fungal organisms seen on GMS and PAS stains	
Ultrasound abdomen	**Mild ascites, mild splenomegaly**	**Large loculated collection in liver with surrounding enhancement**	
Bone marrow aspirate and biopsy (Site: right iliac crest)		**Hypercellular bone marrow with marked myeloid hyperplasia and subset dysmegakaryopoiesis**	
Bone marrow differential (total number of cells counted: 200) *Promyelocyte* *Myeloid* *Erythroid* *Lymphocyte* *Monocyte* *Eosinophil*		1% 73% 19.5% 2.5% 3% 1%	
Genome sequencing		**p.(Glu288Val) (GAA>GTA): c.863 A>T in exon 3 of *SERPINA1* gene**	N/A

^1^Probably hemodilution-induced, transfused packed red cells.

^2^GI PCR: Salmonella, Shigella, E. coli, Cryptosporidium, etc.

WBC, white blood count; ANC, absolute neutrophil count; INR, international normalized ratio; ESR, erythrocyte sedimentation rate; CRP, C-reactive protein; A1AT, alpha-1-antitrypsin; ALT, alanine transferase; GGT, gamma glutamyl transferase; LDH, lactate dehydrogenase; RSV, respiratory syncytial virus; PCR, polymerase chain reaction; PAS, periodic acid-Schiff; PASD, periodic acid-Schiff staining with diastase; GMS, Grocott methenamine silver.

Bold indicates abnormal/significant findings.

**Table 3:** Immune phenotyping.

Immunological tests	Values	Normal ranges
Total IgG	**1,486**	400-1,300 mg/dL
IgG subclass 1 (mg/dL, serum)	612	167–900 mg/dL
IgG subclass 2 (mg/dL, serum)	149	55–359 mg/dL
IgG subclass 3 (mg/dL, serum)	53	34–85 mg/dL
IgG subclass 4 (mg/dL, serum)	11	1–34 mg/dL
IgA	91	20–230 mg/dL
IgE	27	<=30 mg/dL
Neutrophil oxidative index	205	>100
% positive polymorphonuclear cells	95	>95%
Total complement (CH50, U/mL)	41	30–75
Total complement assay *Alternative pathway activity (U/mL, serum)*	88	77–159
CD3+ T cells, absolute	3,117	1,900–6,200 cells/µL
CD3+ CD4+ T cells, absolute	2,407	1,300–3,400 cells/µL
CD3+ CD8+ T cells, absolute	659	620–2,000 cells/µL
CD19+ B cells, absolute	2,003	610–2,600 cells/µL
CD3- CD16+/CD56+ NK cells, absolute	**71**	160–1,100 cells/µL
% CD3+ T cells	60	49-84%
% CD3+ CD4+ T cells	46	28-52%
% CD3+ CD8+ T cells	13	14-30%
% CD19+ B cells	38	13-37%
% CD3- CD16+/CD56+ NK cells	**1**	3-15%
CD4:CD8 ratio	3.60	1.30–3.90
% T-sum	1	
TEMRA CD4+ CD45RA+ CCR7- T cells	0.2	0.2-2.9%
Naïve CD4+ CD45RA+ CCR7+ T cells	91.4	66.3-89.4%
Effector memory CD4+ CD45RA-	1.4	1.3-9.4%
Central memory CD4+ CD45RA-	**7.1**	9.2-22.4%
TEMRA CD8+ CD45RA+ CCR7- T cells	**4.6**	6.4-20.8%
Naïve CD8+ CD45RA+ CCR7+ T cells	81.5	57.8-82.9%
Effector memory CD8+ CD45RA-	12.3	5.1-25.1%
Central memory CD8+ CD45RA-	1.5	1.7-8.5%
CD3+ CD4- CD8- TCR a/b T	0.2	0.2-1.1%
CD3+ TCR g/d T cells	1.1	0.6-12.0%
CD4+ CD25+ CD127+ activated T cells	5.6	3.7-11.1%
CD4+ CD25high CD127low regulatory T cells	7.9	7.7-11.5%
Switched memory IgD- CD27+ B cells	4.0	1.4-11.9%
Unswitched memory IgD+ CD27+ B cells	3.0	3.0-10.7%
Naïve IgD+ CD27- B cells	92.0	76.5-94.7%
Response to *S. pneumoniae* vaccination	10 serotypes >1.3 µg/mL	Good responder: At least a two-fold increase and/or a post-vaccination concentration >1.3 µg/mL

Ig, immunoglobulin; TEMRA, terminally differentiated effector memory T cells re-expressing CD45RA.

Bold indicates abnormal/significant findings.

## Discussion

We report the first known association between *P. aeruginosa* liver infection and homozygosity for the potentially pathogenic S allele of *SERPINA1* gene encoding A1AT. The patient, a previously healthy toddler with a history of brief, self-limited febrile illnesses treated intermittently with antibiotics, presented with persistent fever, anasarca, and dehydration. Genetic testing identified a *SERPINA1* variant found in ~3.6% of individuals in the heterozygous form, with an estimated homozygosity rate of 1 in 600. Although biallelic pathogenic variants in *SERPINA1* can cause autosomal recessive A1ATD predisposing to liver disease, such presentations are uncommon in children (17, 18). Homozygosity for the E288V variant in *SERPINA1* has been only rarely associated with clinically significant lung or liver disease (19).

Identification of the p.Glu288Val variant in the patient’s clinically asymptomatic father highlights incomplete penetrance and variable expressivity, frequently observed in *SERPINA1* mutations. This variant may act as a predisposing genetic factor rather than a primary driver of spontaneous abscess formation. The pathogenicity of the S allele variant is supported by its critical localization within the A1AT protein structure. This substitution occurs in a region conserved across several SERPINs, where the shift from a polar, negatively charged glutamic acid to a non-polar, hydrophobic valine is predicted to disrupt local bond formations. Such biochemical shifts may interfere with proper protein folding or intracellular transport, leading to the retention of misfolded proteins within the endoplasmic reticulum (ER).

The patient’s viral co-infections (RSV and rhinovirus) may have exacerbated the misfolding of A1AT proteins within the hepatocytes. The concurrent infections likely created an immune-compromised state, and the *SERPINA1* variant may have acted as a disease modifier by failing to regulate the resulting protease-driven inflammation, leading to more severe tissue damage as well as depletion of short palate lung and nasal epithelium clone-1 (SPLUNC1), key to respiratory tract host defense against *P. aeruginosa* (20). The lack of chronic cholestasis supports the acute infectious process in this patient, with the liver abscess likely representing an unusual complication of systemic seeding rather than classic A1AT deficiency-related cirrhosis. The atypical presentation of a *Pseudomonas* liver abscess in this otherwise healthy child suggests that homozygosity for the S allele (p.Glu288Val variant) may lower the threshold for hepatic vulnerability, even if it does not result in gross histopathological hallmarks.

A1AT is a potent regulator of neutrophil activation through both protease-inhibitory and non-inhibitory mechanisms (12). Neutrophils serve as the first line of defense against bacterial invasion and coordinate immune responses through interactions with lymphocytes, dendritic cells, and natural killer cells (21, 22). A1AT contributes to antimicrobial defense, and A1AT deficiency has been linked to impaired bacterial clearance (23, 24). Notably, aerosolized A1AT enhances neutrophil-mediated killing of *P. aeruginosa* (23, 25). Experimental studies indicate that A1AT possesses intrinsic anti-*Pseudomonas* properties, and that A1ATD may compromise host defense by permitting NE-mediated inactivation of CXCR1, thereby reducing CXCL-8 binding and attenuating the pro-inflammatory response (12, 25–28).

As with any case report, ours has limitations including representing a single study participant and providing an association without directly demonstrating a mechanism of action. Nevertheless, several lines of evidence suggesting a true association of homozygosity for the S allele (p.Glu288Val variant) of A1AT and our patient’s *P. aeruginosa* infection, including: (a) A1AT serves important host defense functions, including modulating activity of agents important in defense against *P. aeruginosa* – neutrophils and the antimicrobial SPLUNC1 protein; (b) multiple murine studies demonstrate that A1AT specifically promotes SPLUNC1-mediated host defense against *P. aeruginosa* (28–30); (c) infants with S allele have higher risk of respiratory infection in infancy; and (d) patients with bronchiectasis and pathogenic variants of A1AT, including the S allele, demonstrate higher rates of colonization with *P. aeruginosa*. Further research is required to better define the basis for the incomplete penetrance of homozygosity for the relatively common S allele of *SERPINA1* (31).

## Conclusion

Rare infections such as *P. aeruginosa* pyogenic liver abscess in an otherwise healthy child should prompt evaluation for an underlying IEI. Although *SERPINA1* variants are classically associated with pulmonary disease, they can occasionally manifest as liver pathology due to the accumulation of misfolded A1AT within hepatocytes and/or impaired ability to protect SPLUNC1 from degradation by neutrophil elastase. Recognition of this genetic defect in our patient potentially provides a unifying explanation for an atypical and severe infection, highlighting the broader immunomodulatory role of A1AT beyond its hepatic and pulmonary effects.

Identification of the *SERPINA1* defect influenced clinical management by guiding the need for longitudinal follow-up with monitoring of liver function and inflammatory markers, and implementation of measures to prevent pulmonary complications later in life. Early genetic diagnosis of pathogenic variants of A1AT allows for targeted surveillance, genetic counseling of the family, and timely intervention – factors that may significantly improve long-term outcomes.

This case underscores the importance of considering potentially pathogenic A1AT variants in the differential diagnosis of *P. aeruginosa* in children, even in the absence of classic pulmonary or hepatic symptoms. A1ATD screening may especially be helpful in cases of unexplained, severe, or recurrent *P. aeruginosa* infections where a standard immunologic work-up is negative. Broader awareness and early genetic evaluation for pathogenic *SERPINA1* variants in similar presentations can facilitate prompt diagnosis, personalized management, and prevention of long-term complications.

## Data Availability

The original contributions presented in the study are included in the article/supplementary material. Further inquiries can be directed to the corresponding author/s.
